# Exploring the Role of Practical and Emotional Death Preparation in Reducing Emotional Distress Among Family Caregivers of Terminally Ill Cancer Patients: A Multicenter Cross-Sectional Study

**DOI:** 10.3390/cancers17081380

**Published:** 2025-04-21

**Authors:** Boram Kim, Jaemin Kim, Hong Yup Ahn, Sunyoung Park, In Cheol Hwang, So-Jung Park

**Affiliations:** 1 Department of Hospice and Palliative Service, National Cancer Center, Goyang 10408, Republic of Korea; boramkim822@ncc.re.kr; 2 Bucheon Woori Hospital, Bucheon 14466, Republic of Korea; jmkwrs@naver.com; 3 Department of Statistics, Dongguk University, Seoul 04620, Republic of Korea; ahn@dongguk.edu; 4 Department of Psychiatry, National Health Insurance Service Ilsan Hospital, Goyang 10444, Republic of Korea; bechungan@hanmail.net; 5 Department of Family Medicine, Gil Medical Center, Gachon University College of Medicine, Incheon 21565, Republic of Korea

**Keywords:** death preparation, emotional distress, family caregivers, terminal cancer patients, palliative care

## Abstract

**Background/Objectives**: Family caregivers (FCs) often feel unprepared for the death of a terminally ill cancer patient and experience psychological distress. However, the link between FCs’ distress and their preparedness during end-of-life care remains unclear. **Methods**: A multicenter survey across nine inpatient palliative care units included 171 FCs who completed a structured questionnaire. Multivariate logistic regression identified factors associated with anxiety and depression, including emotional and practical preparedness for death. **Results**: Many FCs reported high psychological distress during end-of-life care. Greater practical preparedness was linked to lower odds of severe anxiety (OR: 0.41, 95% CI: 0.27–0.63) and severe depression (OR: 0.51, 95% CI: 0.31–0.85). Although emotional preparedness correlated with distress, this association weakened after adjusting for confounders. **Conclusions**: FCs’ anxiety and depressive symptoms underscore the need for healthcare professionals to provide practical support. Targeted interventions may improve FCs’ preparedness and mitigate psychological distress.

## Simple Summary

Caring for a terminally ill cancer patient can be emotionally overwhelming, especially when family caregivers feel unprepared for their loved one’s passing. This study examined the association between family caregivers’ preparedness for death and their psychological distress by surveying 171 caregivers in inpatient palliative care units. The findings revealed that greater practical preparedness correlated with lower levels of anxiety and depression. These results underscore the vital role of healthcare professionals in equipping family caregivers with guidance and resources to strengthen their practical preparedness for their patient’s death.

## 1. Introduction

Family caregivers (FCs) play a vital role in supporting terminally ill patients with cancer by providing emotional, physical, and practical care throughout the illness trajectory [1]. In Korea, where family-centered caregiving remains prevalent, spouses and adult children serve as primary caregivers for hospice patients, comprising 83.3% of FCs [2]. Providing end-of-life (EOL) care for a loved one imposes a substantial emotional and physical burden on FCs [3]. Studies indicate that a significant proportion of FCs caring for advanced or terminal patients with cancer experience clinically relevant anxiety and depression, with prevalence rates ranging from 38% to 73% [4,5,6,7]. This strain is intensified by the daily demands of caregiving, witnessing patient suffering, and coping with anticipatory grief, all of which contribute to psychological distress [8,9]. Psychological distress in FCs leads to exhaustion, reduced quality of life, impaired caregiving capacity, and worsened patient outcomes, including increased symptom burden and declining health status [5,10].

Death preparedness is a key factor in alleviating this psychological distress among FCs. It includes both practical readiness (e.g., funeral planning and financial arrangements) and emotional preparation (i.e., a subjective sense of readiness to face the patient’s imminent passing). Previous studies have shown that inadequate death preparedness among FCs is linked to heightened psychological distress, elevated anxiety, an increased risk of complicated grief, and difficulties in post-bereavement adjustment [11,12]. These findings underscore the importance of death preparedness as a core component of comprehensive palliative care, potentially fostering caregivers’ emotional resilience during the EOL period.

However, while previous research has primarily focused on bereavement outcomes, such as complicated grief and long-term psychological distress in FCs, limited attention has been given to its impact during the patient’s EOL period [12]. Moreover, many studies have not directly assessed preparedness for death, often relying on tools like the Preparedness for Caregiving Scale (PCS), which measures general caregiving roles rather than death-specific preparation [13,14], or retrospective assessments prone to recall bias [11]. Furthermore, prior studies have largely examined overall preparedness for death, lacking the depth to capture its multifaceted nature [15,16]. Additionally, much of the literature has not specifically targeted FCs of terminally ill patients with cancer, limiting its applicability to this population. These gaps underscore the need for a study that evaluates the relationship between death preparation and emotional distress during the EOL phase, using direct measures of both practical and emotional preparedness. Given the potential for pre-loss distress to influence caregiving experiences and EOL decision-making, understanding this association is crucial. Therefore, this study aims to investigate the relationship between death preparation—categorized into practical and emotional dimensions—and emotional distress among FCs of terminally ill patients with cancer during the patient’s EOL period.

## 2. Materials and Methods

### 2.1. Study Design and Data Collection

This multicenter cross-sectional study was conducted across 9 inpatient palliative care units in South Korea, with data collection spanning September 2021 to May 2023. The study enrolled FCs who were primary relatives providing informal care to terminally ill patients with cancer. Questionnaires were administered consecutively to all eligible FCs. Eligible participants were aged 20 years or older, capable of providing informed consent, and able to complete the self-reported questionnaire. The primary reason for non-participation was the discomfort experienced by FCs. An a priori sample size estimation was not feasible due to the absence of comparable prior studies. Data were collected primarily within one week of patient admission. Researchers and trained assistants explained the study’s purpose, obtained informed consent, and administered self-reported questionnaires. To ensure data completeness, responses were reviewed immediately, and missing data were addressed promptly.

### 2.2. Measurements

#### 2.2.1. Death Preparation

The readiness of FCs for a patient’s death was assessed across emotional and practical dimensions [17]. Emotional preparedness was measured using the statement, “I feel emotionally prepared for the patient’s death”, and practical preparedness with, “I feel practically prepared for the patient’s death, including new responsibilities, future planning, and funeral arrangements”. Responses were rated on a five-point Likert scale: (1) Not at all, (2) No, (3) Moderately, (4) Yes, and (5) Very much so. These two items were independently adopted, separate from the questionnaire items used for the measurement of dependent and covariate variables. This instrument has been utilized in prior palliative care studies focusing on caregivers [12,16,18], and its reliability has been established [19]. Additionally, a panel of palliative care specialists reviewed this instrument for this study to ensure it accurately captured emotional and practical preparedness.

#### 2.2.2. Emotional Distress

Emotional distress was assessed using the 14-item Hospital Anxiety and Depression Scale (HADS), a widely used tool designed to evaluate psychological distress, specifically symptoms of anxiety and depression [20]. It consists of 14 self-report items divided equally into 2 subscales: anxiety (7 items) and depression (7 items). Each item is rated on a four-point Likert scale (0 to 3), with total subscale scores ranging from 0 to 21. Higher scores indicate greater anxiety or depression severity. Originally developed for use in non-psychiatric hospital settings to exclude somatic symptoms, HADS has since been validated in both clinical and non-clinical populations. This scale has been extensively applied in studies assessing psychological distress not only among patients with cancer but also their FCs [21,22]. In this study, FCs’ anxiety and depression symptoms were measured using the Korean version of HADS, which has been validated in the Korean population, with a Cronbach’s alpha of 0.89 for HADS-Anxiety and 0.86 for HADS-Depression [23]. HADS scores were categorized into 3 groups: 0–7 as normal, 8–10 as borderline, and 11 or more as abnormal.

#### 2.2.3. Covariates

Sociodemographic characteristics included age, gender, relationship to the patient, marital status, and religion. Participants’ relationship to the patient was categorized as ‘spouse’ or ‘other’ (including children, siblings, parents, and other relatives). Education level was classified as ‘high school or less’ or ‘college or higher’. Marital status was categorized as ‘married’ or ‘unmarried’ (including never married, divorced, separated, or widowed). Religious affiliation was grouped as ‘no religion’ or ‘professed religion’ (including Protestant, Catholic, Buddhist, and others). FCs’ resilience was assessed using the Connor–Davidson Resilience Scale (CD-RISC) [24], which evaluates individuals’ perceived ability to overcome adversity and persevere. The Korean version used in this study consisted of 25 items [25], with responses rated on a five-point Likert scale ranging from “Not true at all” to “True nearly all of the time”. Higher scores, ranging from 0 to 100, indicated greater resilience. This scale demonstrated excellent internal consistency, with a Cronbach’s alpha of 0.93.

The caregiving environment was examined by assessing social support and family functioning. Social support was measured using the Medical Outcome Study Social Support Survey (MOS-SSS) [26], which was translated into Korean and validated [27], was used to assess social support. This scale exhibited excellent internal consistency, with a Cronbach’s alpha of 0.97. This tool consists of 19 items rated on a five-point scale, ranging from “not at all” to “all of the time”. It evaluates support in the areas of emotional, informational, tangible, affectionate support, and positive social interaction. Higher scores within the range of 0 to 100 reflect a higher level of social support. Family functioning was assessed using the Korean version of the APGAR (Family Adjustment, Partnership, Growth, Affection, and Resolution) tool, which has been validated for reliability in Korea [28]. It consists of 5 items, each rated on a three-point scale: “hardly ever”, “sometimes”, and “almost always”. The total score ranges from 0 to 10, with higher scores reflecting greater satisfaction with family functioning.

### 2.3. Statistical Analysis

The distributions of FCs’ characteristics were summarized as means with standard deviations for continuous variables and frequencies with percentages for categorical variables. Differences in participant characteristics across anxiety and depression categories were analyzed using the Chi-square test for categorical variables and one-way analysis of variance for continuous variables after testing for normality. The relationships between FCs’ emotional and practical preparedness for patient death and their anxiety and depression levels were assessed using Pearson correlation coefficients. Stepwise multivariate logistic regression analyses were conducted, adjusting for covariates (age, gender, relationship to the patient, resilience, social support, and family functioning) to examine the association between death preparedness and emotional distress (anxiety and depression analyzed separately). Variance inflation factors among independent variables were negligible (all <5). Statistical analyses were performed using STATA/MP version 17.0 (StataCorp, College Station, TX, USA), with statistical significance set at *p* < 0.05.

## 3. Result

A total of 171 FCs were included in this study (Table 1). The mean caregiver age was 54.0 years (SD = 13.0), with the majority being female (75.4%). Regarding their relationship with the patient, 40.4% were spouses, and 77.7% were married. Psychosocial characteristics were assessed using validated scales: CD-RISC (mean 58.8, SD = 17.3), MOS-SSS (mean 75.3, SD = 18.0), and the APGAR family function scale (mean 6.2, SD = 2.6). Caregivers’ preparedness for death had mean scores of 3.2 (SD = 1.0) for emotional preparedness and 3.2 (SD = 0.9) for practical preparedness. The mean HADS-Anxiety score was 10.6 (SD = 4.3), while the mean HADS-Depression score was 8.3 (SD = 4.3).

Table 2 presents FC characteristics categorized by anxiety and depression levels. Among those classified for anxiety, 41 (24.0%) were normal, 49 (28.7%) borderline, and 81 (47.4%) abnormal. For depression, 75 (43.9%) were normal, 50 (29.2%) borderline, and 46 (26.9%) abnormal. Significant differences across anxiety levels were found for resilience (*p* < 0.001), social support (*p* = 0.001), family functioning (*p* < 0.001), emotional death preparedness (*p* = 0.014), and practical death preparedness (*p* < 0.001). Similarly, for depression, significant differences were observed in resilience (*p* < 0.001), family functioning (*p* = 0.002), emotional death preparedness (*p* < 0.001), practical death preparedness (*p* < 0.001), and gender (*p* = 0.014), with the proportion of females differing across groups (normal: 69.3%, borderline: 70.0%, abnormal: 91.3%). Social support showed a trend toward significance for depression levels (*p* = 0.063), suggesting a potential association that did not meet the statistical threshold.

Table 3 presents the results of stepwise multivariate logistic regression analyses examining factors associated with borderline and abnormal levels of anxiety and depression. For anxiety, higher resilience (OR = 0.96, 95% CI: 0.93–0.99) and social support (OR = 0.97, 95% CI: 0.94–0.99) were linked to lower odds of borderline anxiety. Practical death preparedness was inversely associated with both borderline anxiety (OR = 0.50, 95% CI: 0.30–0.86) and abnormal anxiety (OR = 0.41, 95% CI: 0.27–0.63). For depression, older age (OR = 0.95, 95% CI: 0.91–0.99) was protective against abnormal depression, while female gender (OR = 8.97, 95% CI: 2.28–35.29) and being a spouse (OR = 3.29, 95% CI: 1.18–9.17) were associated with higher odds of abnormal depression. Greater social support (OR = 0.97, 95% CI: 0.94–0.99) was linked to lower odds of abnormal depression, while better family functioning (OR = 0.83, 95% CI: 0.72–0.96) was associated with lower odds of borderline depression. Practical death preparedness was protective against both borderline depression (OR = 0.83, 95% CI: 0.32–0.96) and abnormal depression (OR = 0.51, 95% CI: 0.31–0.85).

**Figure 1:**
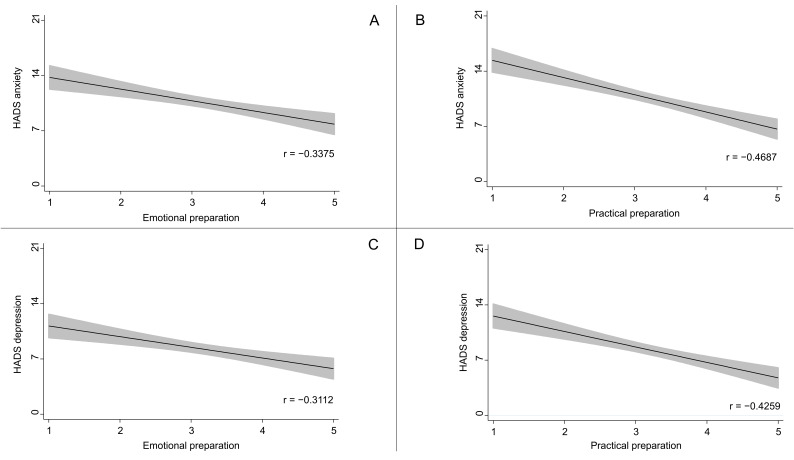
Correlation between death preparation and emotional distress: (**A**) Emotional preparation and HADS anxiety; (**B**) practical preparation and HADS anxiety; (**C**) emotional preparation and HADS depression; (**D**) practical preparation and HADS depression. Note: Presented as correlation coefficient and corresponding 95% confidence interval (all *p* < 0.001; from Pearson correlation analysis). HADS, Hospital Anxiety and Depression Scale.

Negative correlations were observed between death preparedness and emotional distress, as shown in Figure 1 (Pearson correlation analysis). Emotional preparedness was inversely correlated with HADS anxiety (r = −0.3375) and depression (r = −0.3112) scores. Similarly, practical preparedness showed inverse correlations with HADS anxiety (r = −0.4687) and depression (r = −0.4259) scores.

## 4. Discussion

Our study found that a significant proportion of FCs of terminally ill patients with cancer experienced psychological burden, which was linked to multiple factors. A key finding is the association between practical preparedness for the patient’s death and lower emotional distress among FCs.

Our findings have important implications for understanding the FCs of terminal patients with cancer. To our knowledge, this is the first study to establish a significant association between FCs’ practical preparedness for the death of terminal cancer patients and their psychological distress. Previous research has examined FCs’ preparedness and its link to emotional distress [13,29], but these studies focused on caregivers’ perceived readiness for caregiving rather than preparation for death. Some studies have explored the emotional and cognitive aspects of FCs’ preparation for a patient’s death and their relationship with depressive symptoms [30,31]. A prior study underscores the significance of this research, highlighting that the behavioral dimension should be considered separately—along with cognitive and affective dimensions—as a distinct component of the multidimensional framework of FCs’ preparedness for a patient’s death [32]. Recognizing this distinction may help foster FCs’ practical preparation for death and, in turn, reduce their psychological distress.

The EOL period involves considerable uncertainty; however, caregivers’ preparation for a patient’s death cannot be fully understood solely through a medical perspective on illness-related uncertainty [33]. Given the lack of a standardized instrument to assess caregivers’ multidimensional preparedness for death [17], prior studies have investigated the link between overall preparedness and psychological distress. FCs with lower preparedness for the death of a cancer patient experienced more severe grief symptoms during the EOL period [34]. As expected, bereaved caregivers who lacked preparedness exhibited higher levels of depression, anxiety, and prolonged grief symptoms [15,16,35,36,37]. Low preparedness for death can negatively impact caregivers’ psychological health by increasing stress at the time of death and evoking regret over missed opportunities for meaningful time with the patient [36].

Some studies proposed a multidimensional framework encompassing cognitive, emotional, and practical (or behavioral) aspects of FCs’ preparation for a patient’s death [32,33]. One study found that FCs who reported greater emotional and practical preparedness exhibited lower post-loss depression [19], supporting our findings. However, few studies have examined the relationship between these dimensions and FCs’ psychological distress during the EOL period of terminally ill patients with cancer. Some studies reported an inverse association between emotional preparedness and depressive symptoms [30,31], but its relationship with anxiety remains unexplored. Moreover, the impact of practical preparedness on psychological distress among FCs of terminal patients with cancer has yet to be investigated. Our study found an association between better practical preparedness for a patient’s death and lower levels of both depression and anxiety among FCs throughout the EOL care period. These findings emphasize the importance of practical preparedness, which requires active effort, including strategic planning, effective communication, problem-solving, and informed decision-making, along with accepting and adapting to an uncertain future [19,32,33]. Without adequate support, FCs of terminally ill patients may struggle to consider their future beyond immediate caregiving responsibilities and may lack the time and energy for such preparation [19,38]. Throughout the patient’s disease process, regular, meaningful conversations between FCs and healthcare professionals, including discussions about EOL care, are key to establishing proper roles and planning ahead [18]. A prior study identified access to and engagement with healthcare professionals as a key palliative care need for cancer caregivers [1,39]. Healthcare professionals play a crucial role in assessing FCs’ preparedness for a patient’s death and providing targeted interventions to enhance their readiness. While previous studies have suggested an inverse association between emotional preparedness and depressive symptoms, our study found no significant associations between emotional preparedness and symptoms of either depression or anxiety. It is possible that the emotional dynamics involved are more complex, with psychological distress and emotional preparation potentially co-occurring as FCs confront the reality of impending loss [18]. Further research is warranted to clarify these nuanced interrelations and to explore the temporal sequence of these psychological responses.

Previous studies reported a high prevalence of anxiety and depression among FCs of patients with incurable or poor prognosis cancer [40,41], consistent with our findings. Furthermore, our results on emotional distress align with previous research on FCs of palliative care patients [22,42,43,44], in which their mean HADS scores exceeded the normal range. The unpredictable trajectory of terminal-phase cancer, combined with the overwhelming demands of caregiving, complicates FCs’ preparations for the patient’s death and contributes to heightened anxiety and depression [45]. As a consequence of their caregiving role, FCs experience increased emotional distress, which intensifies as the patient’s condition worsens and death approaches [46,47]. Given the strong association between psychological distress in patients with cancer and their caregivers [48], it is essential to consider the patient–caregiver dyad as a treatment unit in clinical approaches [49].

Consistent with previous findings [42,50,51,52,53], our study found that younger, female, and spousal FCs were more likely to report depressive symptoms. Young caregivers may perceive an impending patient death as profoundly distressing, and women tend to assume many caregiving responsibilities [50]. Spousal caregivers often experience greater emotional strain than others, exacerbated by the fact that patients usually share their emotions with their spouses [52]. Moreover, as identified in prior studies [47,51,54], a significant association was found between greater perceived social support and lower levels of psychological distress among FCs. Caregivers with strong social networks can alleviate their care burden by sharing responsibilities, contributing to improved mental health [8]. A prior study indicated that FCs with higher levels of social support demonstrated greater capacity to manage caregiving demands, resulting in lower depression levels while caring for a terminally ill patients with cancer [47]. These findings provide further evidence of the beneficial effects of social support on FCs’ psychological well-being and underscore the need for interventions that enhance social support.

### Limitations

This study has some limitations. First, as this study was cross-sectional, causal relationships could not be determined. Future research using longitudinal designs is needed to examine the temporal dynamics and potential long-term benefits of death preparation on FCs’ mental health. Second, we used a convenience sampling approach by recruiting only FCs who voluntarily consented to participate, which may have introduced selection bias and limited the generalizability of our findings. Furthermore, to minimize recall bias, data were collected using validated questionnaires administered within one week of patient admission; however, self-reported data remain subject to potential inaccuracies. Third, variables previously linked to FCs’ mental health, such as caregiving-related factors (e.g., caregiving burden, financial difficulties, unemployment) and patient-related factors (e.g., symptom distress, poor well-being, low-performance status), were not assessed due to their exclusion from the survey, despite their established associations with increased depression and anxiety [31,50,51,55]. Lastly, self-reported preparedness for death was measured using a Likert scale, which limits a comprehensive understanding of FCs’ actual level of preparation. Although prior research suggests that FCs’ preparation for the death of a patient in EOL care comprises cognitive, emotional, and behavioral dimensions [32], no standardized tool has been developed to assess these aspects. A validated multi-item assessment tool is needed to capture the multidimensional nature of FCs’ preparedness for the death of patients with terminal cancer.

## 5. Conclusions

This study examined the association between death preparation and emotional distress among FCs of terminally ill patients with cancer. The findings suggest that FCs with greater practical preparedness experience lower levels of depression and anxiety, an association observed across various factors, highlighting its significance to FCs’ psychological well-being. The results underscore the essential role of healthcare professionals in providing guidance and enhancing FCs’ capacity for preparation. Further research is needed to explore how caregivers practically prepare for a patient’s death and to determine the most effective timing and methods for delivering support.

## Figures and Tables

**Table 1:** Characteristics and distribution of scores (*n* = 171).

		Range	
	Mean ± SD or *n* (%)	Interquartile	Possible
Demographics			
Age (years)	54.0 ± 13.0	44–62	
Sex (female)	129 (75.4)		
Spouse	69 (40.4)		
Education (≥college)	94 (55.3)		
Married	132 (77.7)		
Religious affiliation	95 (56.2)		
Resilience (CD-RISC)	58.8 ± 17.3	47–70	0–100
Social support (MOS-SSS)	75.3 ± 18.0	61–90	0–100
Family function (APGAR)	6.2 ± 2.6	5–8	0–10
Preparedness for death			
Emotional	3.2 ± 1.0	2–4	1–5
Practical	3.2 ± 0.9	3–4	1–5
Emotional distress			
HADS-Anxiety	10.6 ± 4.3	8–14	0–21
HADS-Depression	8.3 ± 4.3	5–11	0–21

CD-RISC, Connor–Davidson Resilience Scale; MOS-SSS, Medical Outcome Study Social Support Survey; APGAR, Adaptation, Partnership, Growth, Affection, and Resolve; HADS, Hospital Anxiety and Depression Scale; SD: Standard Deviation.

**Table 2:** Characteristics related to emotional distress ^a^.

	Anxiety Level			*p*-Value	Depression Level			*p*-Value
	Normal	Borderline	Abnormal		Normal	Borderline	Abnormal	
Number	41	49	81		75	50	46	
Demographics								
Age (years)	55.8 ± 13.3	56.4 ± 11.0	51.7 ± 13.7	0.086	55.4 ± 13.0	54.9 ± 12.2	50.7 ± 13.5	0.135
Sex (female)	32 (78.1)	32 (65.3)	65 (80.3)	0.144	52 (69.3)	35 (70.0)	42 (91.3)	0.014
Spouse	12 (29.3)	21 (42.9)	36 (44.4)	0.249	24 (32.0)	22 (44.0)	23 (50.0)	0.121
Education (≥college)	28 (68.3)	25 (51.0)	41 (51.3)	0.158	47 (63.5)	26 (52.0)	21 (45.7)	0.137
Married	33 (80.5)	42 (85.7)	57 (71.3)	0.141	60 (81.1)	39 (78.0)	33 (71.7)	0.489
Religious affiliation	27 (65.9)	28 (57.1)	40 (50.6)	0.277	43 (58.1)	31 (62.0)	21 (46.7)	0.293
Resilience (CD-RISC)	70.5 ± 16.3	55.4 ± 15.5	54.8 ± 16.3	<0.001	64.3 ± 17.2	57.4 ± 14.3	51.7 ± 17.8	<0.001
Social support (MOS-SSS)	84.2 ± 12.9	72.4 ± 17.1	72.4 ± 19.4	0.001	78.5 ± 16.1	74.5 ± 16.2	70.5 ± 21.9	0.063
Family function (APGAR)	7.5 ± 2.4	6.2 ± 2.2	5.6 ± 2.8	<0.001	6.9 ± 2.4	6.3 ± 2.3	5.2 ± 3.0	0.002
Preparedness for death								
Emotional	3.5 ± 0.9	3.2 ± 0.9	2.9 ± 1.0	0.014	3.4 ± 0.9	3.2 ± 1.0	2.7 ± 1.0	<0.001
Practical	3.7 ± 0.7	3.4 ± 0.8	2.8 ± 0.9	<0.001	3.6 ± 0.8	3.2 ± 0.8	2.6 ± 1.0	<0.001

CD-RISC, Connor–Davidson Resilience Scale; MOS-SSS, Medical Outcome Study Social Support Survey; APGAR, Adaptation, Partnership, Growth, Affection, and Resolve. Data are presented as mean (standard deviation) or number (percentage). ^a^ Assessed using a Hospital Anxiety and Depression Scale (in subscale: ≤7, normal; 8–10, borderline; and ≥11, abnormal).

**Table 3:** Odds ratios ^a^ and 95% confidence interval for emotional distress ^b^ of family caregivers.

	Anxiety		Depression	
	For Borderline	For Abnormal	For Borderline	For Abnormal
Old age (per 1-y increase)				0.95 (0.91–0.99) **
Female				8.97 (2.28–35.29) **
Spouse			2.07 (1.02–4.18) *	3.29 (1.18–9.17) *
Highly resilient (per 1-pt increase)	0.96 (0.93–0.99) **			
Good social support (per 1-pt increase)	0.97 (0.94–0.99) *	0.98 (0.96–1.00) *		0.97 (0.94–0.99) *
Functional family (per 1-pt increase)			0.83 (0.72–0.96) *	
Well prepare practically (per 1-pt increase)	0.50 (0.30–0.86) *	0.41 (0.27–0.63) ***	0.83 (0.32–0.96) **	0.51 (0.31–0.85) *

Only significant variables are presented (* *p* < 0.05; ** *p* < 0.01; *** *p* < 0.001). ^a^ From stepwise multivariate logistic regression models including all variables in Table 1. ^b^ Assessed using a Hospital Anxiety and Depression Scale (in subscale: ≤7, normal; 8–10, borderline; and ≥11, abnormal).

## Data Availability

The data presented in this study are available upon request from the corresponding authors; they are not publicly available due to privacy concerns related to research participants.

## Abbreviations

The following abbreviations are used in this manuscript:

FCs	Family caregivers
EOL	End-of-life
HADS	Hospital Anxiety and Depression Scale
